# Echocardiographic Grading of Right Ventricular Afterload in Left Heart Disease: Relation to Right Ventricular Function, Pulsatile and Resistant Load, and Outcome

**DOI:** 10.1002/pul2.70055

**Published:** 2025-02-20

**Authors:** Bettia Edith Celestin

**Affiliations:** ^1^ Division of Cardiology, Department of Medicine Stanford University School of Medicine Stanford California USA; ^2^ Division of Pathology, Department of Medicine Stanford University School of Medicine Stanford California USA; ^3^ Cardiovascular Institute Stanford University School of Medicine Stanford California USA

**Keywords:** assessment, noninvasive, pulmonary hypertension, severity

In the study “Echocardiographic grading of right ventricular afterload in left heart disease: relation to right ventricular function, pulsatile and resistant load, and outcome,” Bech‐Hanssen et al. addressed an interesting topic about severity gradation using right ventricular (RV) afterload echocardiographic assessment. In a population of group 2 pulmonary hypertension (PH), resistant and pulsatile RV afterload metrics were incorporated in an original score based on echocardiographic doppler analysis.

In daily clinical practice, pulmonary vascular resistance, is commonly measured using invasive technique during right heart catheterization in severe patients. However, as the authors mentioned, the RV afterload has resistive and pulsative component [1]. The overlooked pulsative component assesses the ability of the pulmonary artery to stretch and expand in response to pressure applied on the pulmonary artery wall. As we know, the pulsative and resistive component of RV afterload have an inverse hyperbolic relationship [2]. Thus, different RV afterload phenotypes can be defined. This novel RV afterload score is based on four echo Doppler parameters with three levels of severity: low, intermediate, and high RV afterload (Figure 1).

**Figure 1:**
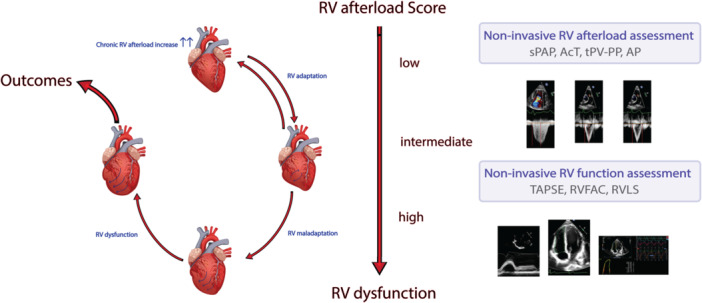
RV afterload to RV dysfunction noninvasive assessment. AcT = acceleration time, AP = augmented pressure, sPAP = systolic pulmonary artery pressure, tPV‐PP = time interval from peak velocity in the RVOT to peak velocity (=peak pressure).

The authors demonstrated well that an intermediate to a high RV afterload score was associated to RV dysfunction (using FAC and RVLS) and low RVD score but also incremented the predictive risk of outcomes beyond NtproBNP, LVEF and NYHA.

This score, focus on RV afterload, is a novelty in the field. In the literature, echocardiographic scores for PH severity have been developed for RV dysfunction [3, 4]. Using RV afterload noninvasive assessment is crucial and may help us to detect early stage of disease before the development of RV dysfunction. The next challenge for future studies will be to explore this score on earlier stage patients and be able to predict RV dysfunction and outcomes for this earlier stage population who remains clinically challenging for diagnosis.

## Author Contributions

The author is solely responsible for the conceptualization, writing, and design of the figure included in this editorial.

## Ethics Statement

The author has nothing to report.

## Conflicts of Interest

The author declares no conflicts of interest.

## Data Availability

The author has nothing to report.
